# Lipogems Product Treatment Increases the Proliferation Rate of Human Tendon Stem Cells without Affecting Their Stemness and Differentiation Capability

**DOI:** 10.1155/2016/4373410

**Published:** 2016-01-06

**Authors:** Pietro Randelli, Alessandra Menon, Vincenza Ragone, Pasquale Creo, Sonia Bergante, Filippo Randelli, Laura De Girolamo, Umberto Alfieri Montrasio, Giuseppe Banfi, Paolo Cabitza, Guido Tettamanti, Luigi Anastasia

**Affiliations:** ^1^IRCCS Policlinico San Donato, San Donato Milanese, 20097 Milan, Italy; ^2^Department of Biomedical Sciences for Health, University of Milan, 20133 Milan, Italy; ^3^IRCCS Istituto Ortopedico Galeazzi, 20161 Milan, Italy; ^4^Università Vita-Salute San Raffaele, 20132 Milan, Italy

## Abstract

Increasing the success rate of rotator cuff healing remains tremendous challenge. Among many approaches, the possibility of activating resident stem cells in situ, without the need to isolate them from biopsies, could represent valuable therapeutic strategy. Along this line, it has been recently demonstrated that lipoaspirate product, Lipogems, contains and produces growth-factors that may activate resident stem cells. In this study, human tendon stem cells (hTSCs) from the rotator cuff were cocultured in a transwell system with the Lipogems lipoaspirate product and compared to control untreated cells in terms of cell proliferation, morphology, stem cell marker and VEGF expression, and differentiation and migration capabilities. Results showed that the Lipogems product significantly increases the proliferation rate of hTSCs without altering their stemness and differentiation capability. Moreover, treated cells increase the expression of VEGF, which is crucial for the neovascularization of the tissue during the healing process. Overall, this study supports that directly activating hTSCs with the Lipogems lipoaspirate could represent a new practical therapeutic approach. In fact, obtaining a lipoaspirate is easier, safer, and more cost-effective than harvesting cells from tendon or bone marrow biopsies, expanding them in GMP facility and then reinjecting them in the patient.

## 1. Introduction

Rotator cuff tears represent the vast majority of shoulder injuries in adult patients and are a common contributing factor to shoulder pain and occupational disability, whose prevalence is rising due to the increase of the world population age [1]. Although surgical procedures for rotator cuff repair have evolved and improved over the past decades, a high rate of retear is still observed [2–4]. This is mainly caused by a failure of tendon healing, especially in the case of the supraspinatus tendon [5–9]. Therefore, increasing the success rate of rotator cuff healing remains a tremendous challenge for orthopedic surgeons, which encourages the development of new alternative therapies [10–15]. In fact, once injured, tendons do not completely regain the normal structural and biomechanical properties, resulting in the formation of scar tissue, adhesions, fatty infiltration, and matrix disorganization, which increase the risk of retear. Among several factors, tendon poor vascularization reduces the availability of oxygen, growth-factors, and other nutrients necessary for tissue regeneration and significantly affects the quality and speed of the tendon healing response. Therefore, in considering new strategies for tendon engineering, the goal of promoting neoangiogenesis is vital to accelerate the healing process. Moreover, several studies have shown that different types of stimuli could activate the normal growth-factor-mediated healing cascades [16]. Along this line, it has been recently demonstrated that lipoaspirates contain and produce growth-factors, such as platelet-derived growth-factor (PDGF), fibroblast growth-factor (FGF), transforming growth-factor beta (TGF-*β*), and vascular endothelial growth-factor (VEGF), which are known to play important regulatory roles in cellular functions, including adhesion, chemotaxis, proliferation, migration, matrix synthesis, differentiation, and angiogenesis [16–18]. The cell fraction responsible for growth-factor production and regulation is mainly the stromal vascular fraction, which could have acceleratory effect on the healing process of injured tendons [19]. Given the tissue availability, as well as the easy and minimally invasive access to tissue sources, adipose tissue may in fact represent a potential choice for tendon repair and regeneration [20]. Among many approaches, an innovative technology, Lipogems, provides a nonexpanded, ready-to-use, and microfragmented adipose tissue that is expected to have the peculiar advantage of maintaining an intact stromal vascular niche harboring cellular elements with mesenchymal stem cell and pericyte characteristics [21]. This system works through a mild mechanical tissue cluster-size reduction in a completely closed system, avoiding the use of any enzyme, additives, and other additional manipulations (i.e., centrifugation and subfractional harvesting) [21].

In a previous study on the Lipogems product, Bianchi et al. speculated that the action of the Lipogems product, due to an intact stromal vascular niche, could involve the secretion of trophic mediators delivering instructive messages that functionally promote a more compliant regenerative environment within the recipient tissue [21]. In particular, the lipoaspirate could stimulate the self-healing processes by activating resident stem cells through paracrine mechanisms. Therefore, the Lipogems product may be instrumental for activating resident progenitor cells to proliferate and contribute to the tendon healing process. Actually, it has been recently demonstrated that also the human rotator cuff contains a reservoir of progenitor cells, which can be isolated and expanded* in vitro* [22–26]. Thus, the availability of a completely closed disposable system for adipose tissue processing could allow the procedure to be readily, safely, and economically performed in clinical settings to afford significant tissue repair, overcoming the difficulty of an* ex vivo* expansion and the complexity of the current Good Manufacturing Practice (GMP) requirements for preparing cells for a therapeutic use [27].

On these bases, the aim of this study was to assess the effects of the Lipogems product on primary cultures of human tendon stem cells (hTSCs).

## 2. Materials and Methods

### 2.1. Cell Cultures

Human tendon stem cells (hTSCs) were isolated from supraspinatus tendon specimens collected during arthroscopic rotator cuff repair, according to our previous procedure [22]. The isolated hTSCs were cultured in* normal growth medium* composed of *α*-Minimal Essential Medium (*α*-MEM) (Sigma-Aldrich) supplemented with 2 mM glutamine (Euroclone), 1% antibiotic-antimycotic mixture (Euroclone), and 20% (v/v) fetal bovine serum (FBS) (HyClone, Thermo Fisher Scientific) at 37°C in a humidified atmosphere of 5% CO_2_. The medium was changed every 2-3 days. All experiments were carried out with cells at passage four after isolation.

### 2.2. Processing of Adipose Tissue with the Lipogems Device

Human subcutaneous adipose tissue samples were obtained with patient informed consent from abdominal lipoaspiration procedures performed before arthroscopic rotator cuff repair and processed by using the Lipogems device, according to the manufacturer's instructions previously described [21]. Avoiding the presence of air, the lipoaspirate tissue was subjected to a first cluster reduction, obtained by pushing the lipoaspirate from the syringe into the device through the first large filter (blue cap), and allowing the corresponding quantity of saline to exit towards the wasting bag. The five stainless steel beads contained in the device were essential to obtain a temporary emulsion between oil, blood, and saline, which could be washed away against density following the current of saline moved by gravity in the wasting bag. After this washing step (the flowing solution appears clear and the lipoaspirate yellow), the saline flux was stopped and the device was reversed (gray cap up), leading to the second adipose cluster reduction. Such reduction was obtained by pushing the floating adipose clusters through the second cutting hexagonal filter (grey cap), pushing fluid from below with a 60 mL syringe. The reduced cluster was collected in another 60 mL syringe placed above and positioned to gently decant the Lipogems product by gravity in order to remove excessive saline solution. A mean of 50 mL of lipoaspirate was collected and processed with the Lipogems device to obtain an amount of 20 mL of the final Lipogems product, which was transferred to several 10 mL syringes to be reinjected in the same patient or used in our experiments.

### 2.3. Lipogems Coculture Experiments of hTSCs with the Lipogems Product

**Figure 1:**
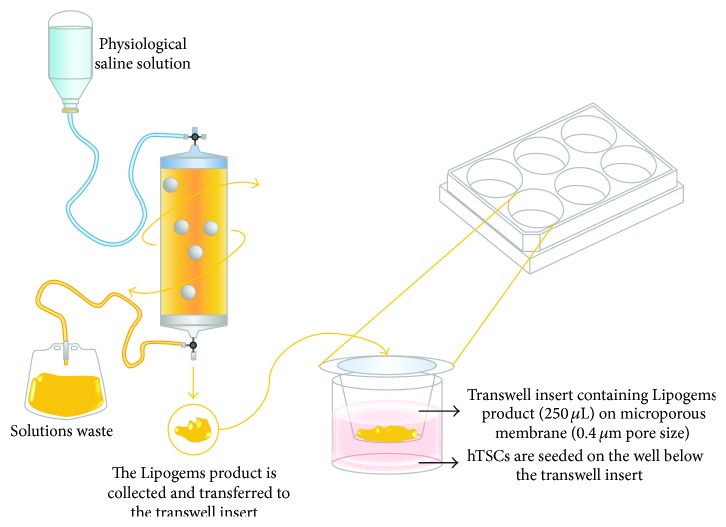
Schematic illustration of the Lipogems device and the transwell coculture system of hTSCs with the Lipogems product. The lipoaspirate was processed using the Lipogems device to obtain the final Lipogems product. A transwell coculture system was used to study the effects of the Lipogems product on primary cultures of hTSCs. Cells were seeded on the bottom of a 6-well plate in normal growth medium. Twenty-four hours after plating, the Lipogems product was transferred in the upper well of a transwell cell culture system (250 *μ*L/transwell insert), separated from hTSCs in the lower well by a 0.4 *μ*m microporous polycarbonate membrane. hTSCs and Lipogems product were maintained in coculture for successive analyses.

hTSCs were seeded on the bottom of 6-well plates in normal growth medium. Twenty-four hours after seeding, a volume of 250 *μ*L of the freshly processed Lipogems product was transferred to the transwell polycarbonate microporous inserts (0.4 *μ*m membrane pore size, Falcon BD, Figure 1) above hTSCs monolayers, allowing for potential diffusion of soluble mediators but preventing cell-cell contact. Control cells were cultured under the same conditions without the Lipogems product. The medium was replaced every 48 h in all experiments. To study the effects of the Lipogems treatment on multilineage differentiation potential of hTSCs, cells were cultured with the Lipogems product in osteogenic and adipogenic differentiation media, as described below.

### 2.4. Cell Morphology and Proliferation

For analysis of cell viability, hTSCs were plated at a concentration of 2.6 × 10^3^ cells/cm^2^ and exposed to the Lipogems product for 96 h, as described above. Cell morphology was examined daily with a phase-contrast microscope (Axiovert 40 CFL, Zeiss, equipped with a Moticam 2300 camera, Motic) to assess the effects of the Lipogems product on hTSCs phenotype. Cell growth curves were prepared by harvesting with Trypsin-EDTA solution (Sigma-Aldrich) and then counting with a Countess Cell Counter (Invitrogen, Life Technologies), according to the manufacturer's procedure. Cell viability was determined by trypan blue dye exclusion assay. All assays were carried out in triplicate for each sample.

### 2.5. Cell Apoptosis

Apoptosis was measured by flow cytometry on Lipogems-treated and control cells at 0, 48, and 96 h of treatment using Annexin V-FITC Apoptosis Detection Kit (Enzo Life Sciences), according to the manufacturer's protocol. Briefly, adherent cells were trypsinized, washed in PBS by gentle shaking, and resuspended with 200 *μ*L of a specific Binding Buffer (10 mM HEPES/NaOH, pH 7.4; 140 mM NaCl; 2.5 mM CaCl_2_) containing 5 *μ*L of Annexin V-FITC. After incubation for 10 min in the dark at room temperature, cells were washed in PBS, resuspended in 200 *μ*L of Binding Buffer, and then stained with 10 *μ*L Propidium Iodide (20 *μ*g/mL). Samples were acquired with a Navios Flow Cytometer (Beckman Coulter) and analyzed using Kaluza 1.2 software (Beckman Coulter).

### 2.6. Flow Cytometric Analysis

After a four-day treatment, Lipogems-treated and control cells were characterized by flow cytometry for the expression of key stem cell markers. Flow cytometry analysis was performed on 1 × 10^5^ cells/sample. Briefly, aspecific binding sites were blocked with a blocking solution (50% 1x PBS, 50% FBS) for 30 min at room temperature and washed twice with PBS at 4°C. Cells were stained with fluorochrome-conjugated mouse antihuman antibodies at the optimal concentration (1 : 20 dilution) in PBS for 10 min at 4°C and washed twice with PBS at 4°C. Cell characterization was performed using the following antibodies: *α*CD9 FITC, *α*CD73 FITC, *α*HLA-DR FITC, *α*CD13 PE, *α*CD29 PE, *α*CD44 PE, *α*CD45 PE, *α*CD71 PE, *α*CD90 PE, *α*CD105 PE, *α*CD106 PE, *α*CD34 PerCP-eFluor710, *α*CD166 PerCP-eFluor710, *α*HLA-ABC FITC, *α*NG2 PE, and *α*SSEA-4 PE (all from eBioscience); *α*Lineage Cocktail FITC, *α*CD18 PE, *α*CD140a PE, *α*CD140b APC, *α*CD146 PE, and *α*Stro-1 Alexa Fluor 647 (all from BioLegend); and *α*CD117 PE (Miltenyi Biotec). The respective isotype antibodies were used as controls. Samples were acquired with a Navios Flow Cytometer (Beckman Coulter) and data were processed with Kaluza software (Beckman Coulter).

### 2.7. Adipogenic Differentiation

To assess the effects of the Lipogems product on the adipogenic differentiation capacity of hTSCs, cells were plated at a concentration of 3 × 10^4^ cells/cm^2^ and preconditioned with the Lipogems product for 96 h in normal growth medium and then switched to DMEM-low glucose (Sigma-Aldrich), 10% FBS (HyClone, Thermo Fisher Scientific), 4 mM L-glutamine (Euroclone), and 1% antibiotic-antimycotic mixture (Euroclone), with the addition of the mesenchymal stem cell adipogenesis kit (Millipore) for 21 days, according to the manufacturer's instructions. At day 21, Oil Red O solution (Millipore) was used to stain lipid droplets of derived adipocytes, according to the manufacturer's procedures. All photomicrographs were acquired with Axiovert 40 microscope (Zeiss) equipped with a Moticam 2300 camera (Motic). The adipogenic medium was changed every 2-3 days.

### 2.8. Osteogenic Differentiation

To assess the effects of the Lipogems product on the osteogenic differentiation capacity of hTSCs, cells were plated at a concentration of 3 × 10^4^ cells/cm^2^ and preconditioned with the Lipogems product for 96 h in normal growth medium and then switched to the osteogenesis induction medium, which was constituted of DMEM-low glucose (Sigma-Aldrich), 10% FBS (HyClone, Thermo Fisher Scientific), 4 mM L-glutamine (Euroclone), and 1% antibiotic-antimycotic mixture (Euroclone), supplemented with 0.1 *μ*M dexamethasone, 50 *μ*g/mL L-ascorbic acid-2-phosphate, and 10 mM *β*-glycerophosphate (all reagents from Sigma-Aldrich) for 17 days. At day 17, Alizarin Red solution (Millipore) was used to detect calcium deposition in derived osteoblasts according to the manufacturer's instruction. All photomicrographs were acquired with Axiovert 40 microscope (Zeiss) equipped with a Moticam 2300 camera (Motic). The osteogenic medium was changed every 2-3 days.

### 2.9. RNA Extraction and Real-Time PCR

Total RNA was isolated using TRIzol Reagent (Ambion, Life Technologies), and 1 *μ*g of extracted RNA was reverse transcribed to cDNA using the iScript cDNA synthesis kit (BioRad) according to the manufacturer's instructions. Real-Time PCR was performed in a 96-well plate with 10 ng of cDNA as template, 0.2 *μ*M primers, and 1x Power SYBR Green PCR Master Mix (Applied Biosystems, Life Technologies) in 20 *μ*L final volume per well using a StepOnePlus Real-Time PCR System (Applied Biosystems). The mRNA levels of Tenomodulin (TNMD), collagen type I alpha-1 (COL1A1), Tenascin C, Nanog homeobox (Nanog), octamer-binding transcription factor 4 (Oct4), kruppel-like factor 4 (KLF4), vascular endothelial growth-factor (VEGF), peroxisome proliferator-activated receptor-*γ* (PPAR-*γ*), lipoprotein lipase (LPL), alkaline phosphatase (ALP), myogenin (MYOG), and myogenic differentiation 1 (MYOD) were assessed. Ribosomal protein S14 (S14) was used as reference gene in quantitative analysis. We performed a series of validation experiments confirming that S14 is constitutively and stably expressed in all cell samples, with an amplification efficiency of 97.8% and linear response in the expression range studied (Supplementary Figure  1 in Supplementary Material available online at http://dx.doi.org/10.1155/2016/4373410). Primer sequences are as follows: TNMD, forward 5′-TGTATTGGATCAATCCCACTCTAAT-3′ and reverse 5′-TTTTTCGTTGGCAGGAAAGT-3′; COL1A1, forward 5′-GGGATTCCCTGGACCTAAAG-3′ and reverse 5′-GGAACACCTCGCTCTCCA-3′; Tenascin C, forward 5′-CGGGGCTATAGAACACCAGT-3′ and reverse 5′-AACATTTAAGTTTCCAATTTCAGGTT-3′; Nanog, forward 5′-GGTCCCAGTCAAGAAACAGA-3′ and reverse 5′-GAGGTTCAGGATGTTGGAGA-3′; Oct4, forward 5′-AGGAGAAGCTGGAGCAAAA-3′ and reverse 5′-GGTCGAATACCTTCCCAAA-3′; KLF4, forward 5′-GACTTCCCCCAGTGCTTC-3′ and reverse 5′-CGTTGAACTCCTCGGTCTC-3′; VEGF, forward 5′-CAACATCACCATGCAGATTATGC-3′ and reverse 5′-TCGGCTTGTCACATTTTTCTTGT-3′; PPAR-*γ*, forward 5′-TTCCTTCACTGATACACTGTCTGC-3′ and reverse 5′-GGAGTGGGAGTGGTCTTCCATTAC-3′; LPL, forward 5′-AGAGAGAACCAGACTCCAATG-3′ and reverse 5′-GGCTCCAAGGCTGTATCC-3′; ALP, forward 5′-CGCACGGAACTCCTGACC-3′ and reverse 5′-GCCACCACCACCATCTCG-3′; MYOG, forward 5′-AAGAAGGGGAGAGGAACAGC-3′ and reverse 5′-GCAACTTCAGCACAGGAGAC-3′; MYOD, forward 5′-GCTAGGTTCAGCTTTCTCGC-3′ and reverse 5′-CACCTGCTACATTTGGGACC-3′; and S14, forward 5′-GTGTGACTGGTGGGATGAAGG-3′ and reverse 5′-TTGATGTGTAGGGCGGTGATAC-3′.

*Amplification Protocol*. An initial denaturation at 95°C for 3 min, followed by 40 cycles of 5 s each at 95°C and 30 s at 57°C. Relative quantification of target genes was performed in triplicate, analyzed using the 2^−ΔΔCt^ method, and normalized to the corresponding S14 values.

### 2.10. Cell Migration by Wound-Healing Assay

Wound-healing assay was performed as previously described [28]. hTSCs were grown to confluence in 6-well plates and treated with the Lipogems product or cultured in the growth medium alone. A sterile P200 pipet tip was used to create a scratch across the cell monolayer. Then, cultures were washed once with 1 mL of growth medium to remove the damaged and detached cells. After replacing the medium, hTSCs were allowed to grow for 48 h. At different time points, cell cultures were examined with a phase-contrast microscope (Axiovert 40 CFL, Zeiss, equipped with a Moticam 2300 camera, Motic) and images of the same scratch fields were acquired at time 0 and after 17, 20, 25, 30, and 42 h from the scratch. The gap area between the cells was calculated in each acquired image using software ImageJ. The migration rate was based on the measure of the recovered wound area (experimental data expressed in percentage). All assays were carried out in triplicate for each sample.

### 2.11. Statistical Analysis

Statistical analysis was performed using GraphPad Prism v 6.0 software (GraphPad Software Inc.). Data were typical results from three replicate experiments for each of the three patients-derived cell lines and were expressed as mean ± standard deviation (SD). Paired comparisons were performed by two-tailed *t*-test. The significance level was set at *p* value lower than 0.05.

## 3. Results

To assess the effects of the Lipogems product on hTSCs, cells were cocultured for up to 96 h with freshly obtained Lipogems product in a transwell cell culture system as described in the Methods and graphically outlined in Figure 1. Control hTSCs were cultured using the same transwell system, but without adding the Lipogems product.

### 3.1. Effects on Cell Morphology and Proliferation

**Figure 2:**
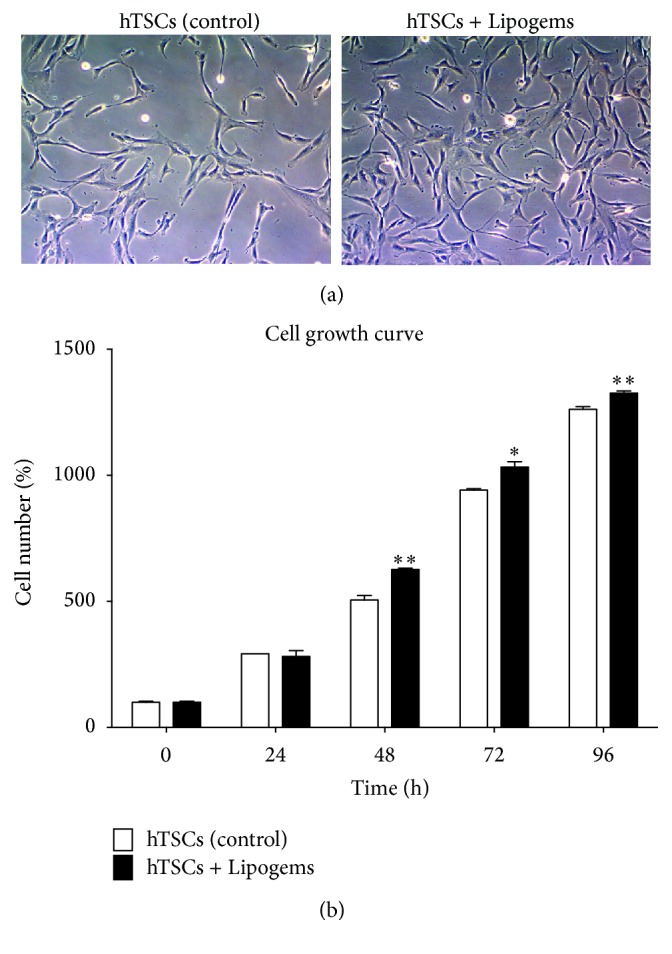
Effects of the Lipogems product on hTSCs morphology and proliferation. (a) Phase-contrast microphotographs (original magnification ×10) and (b) cell growth curves of hTSCs during 96 h treatment with the Lipogems product in normal growth medium and compared to control cells. All experiments were performed in triplicate. Error bars show the mean ± SD of three different experiments. *p* values were calculated using Student's *t*-test. Only *p* values < 0.05 are indicated: ^*∗*^ *p* < 0.05; ^*∗∗*^ *p* < 0.01, as compared to control cells.

Cell morphology analysis revealed no significant differences between Lipogems-treated and control cells during 96 h treatment (Figure 2(a), cell morphology at 48 h after treatment). Cell growth analysis revealed a statistically significant increase in cell proliferation of Lipogems-treated cells as compared to controls, the effect being statistically significant after 48 h. In particular, hTSCs showed a significant increase of 24% (*p* < 0.01), 9.7% (*p* < 0.05), and 5% (*p* < 0.01) in the presence of the Lipogems product at 48, 72, and 96 h, respectively, as compared to control cells (Figure 2(b)).

### 3.2. Effects on Cell Apoptosis

**Figure 3:**
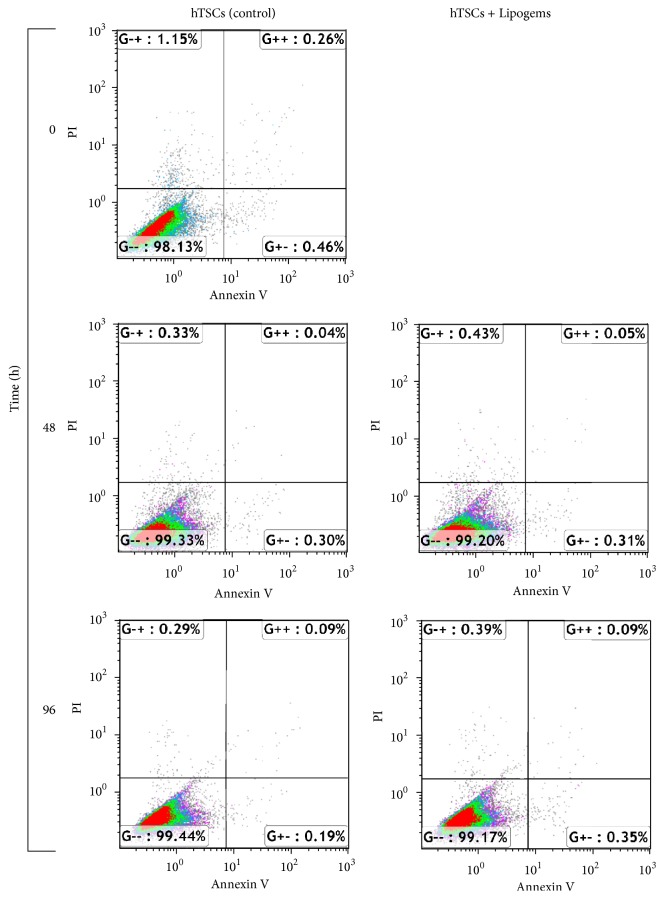
Effects of the Lipogems product on hTSCs apoptosis. Flow cytometric analysis of hTSCs survival rate at 0, 48, and 96 h after the treatment with the Lipogems product (right panel) as compared to control cells (left panel), through double staining with Annexin V-FITC and PI. Early apoptotic cells (Annexin V-positive/PI-negative) are localized in the lower right region, late apoptotic and necrotic cells (Annexin V-positive/PI-positive) are localized in the upper regions, and vital cells (double negative) are localized in the lower left region.

Apoptosis was measured by flow cytometry on hTSCs at 0, 48, and 96 h of treatment with the Lipogems product using Annexin V-FITC and compared to control cells at the same time points (Figure 3). Results revealed no significant apoptosis in all tested samples (always below 0.5%), with no significant differences between control and Lipogems-treated cells.

### 3.3. Effects on Cell Phenotype by Flow Cytometry

The expression of key stem cell markers was evaluated by flow cytometry in hTSCs after a 96 h treatment with the Lipogems product and compared to control untreated cells. Results of comparative flow cytometry analyses showed no significant differences between the two groups for all tested markers (Table 1).

### 3.4. Effects on Tenogenic, Adipogenic, Osteogenic, and Myogenic Differentiation Promotion

**Figure 4:**
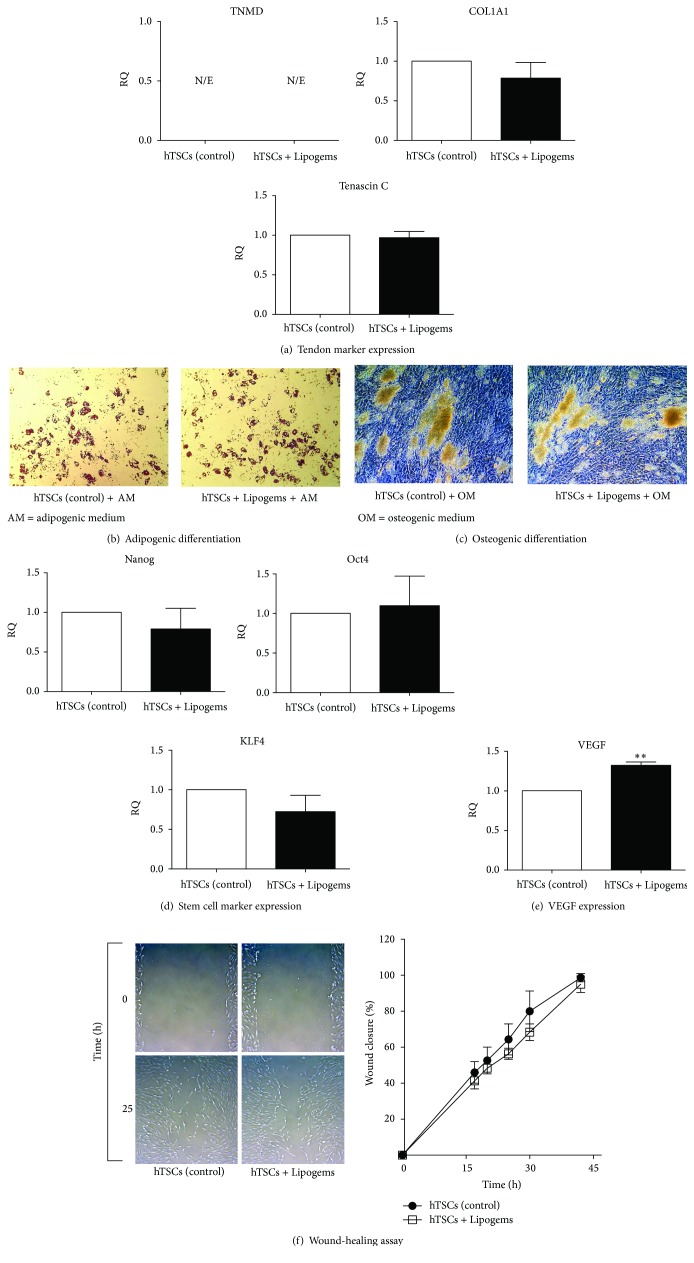
Effects of the Lipogems product on tendon marker expression. (a) Gene expression of TNMD, Tenascin C, and COL1A1 by Real-Time PCR at 96 h of treatment with the Lipogems product. Values are expressed as fold-changes relative to control cells. Effects of the Lipogems product on the* in vitro* differentiation of hTSCs toward the adipogenic and the osteogenic phenotypes. Adipogenic and osteogenic differentiation ability of hTSCs cultured with the Lipogems product in the appropriate differentiation medium was evaluated by Oil Red O (b) and Alizarin Red-S (c) staining, respectively. (b) Lipid intracellular droplets (red) in the adipocytes were stained with Oil Red O solution. (c) Alizarin Red-S staining revealed the presence of calcium deposits (yellowish-brown). Typical results are shown. Original magnification ×10. Effect of Lipogems treatment on stem cells marker (Nanog, Oct4, and KLF4) (d) and VEGF (e) expression by Real-Time PCR at 96 h of treatment with the Lipogems product. Values are expressed as fold-changes relative to control cells. Effect of the Lipogems product on hTSCs migration. (f) Representative time-lapse migration images of control and Lipogems-treated cells. Images were acquired at 0 and 25 h in* in vitro* wound-healing assay. Original magnification ×5. The migration rate was measured by quantifying the total area of the wounded region lacking cells. The average percentages of recovered area obtained from three different experiments at 17, 20, 25, 30, and 42 h of treatment with the Lipogems product, as compared to control cells. All quantitative data are expressed as the means ± SD of three different experiments. *p* values were calculated using Student's *t*-test. Only *p* values < 0.05 are indicated: ^*∗∗*^ *p* < 0.01, compared to control cells.

We assessed whether Lipogems product treatment would induce hTSCs to differentiate into other cell phenotypes. We found that 96 h exposure to Lipogems product did not increase nor induce the expression of mRNA levels of tenogenic (Tenomodulin, COL1A1, Tenascin C) (Figure 4(a)), adipogenic (PPAR-*γ* and LPL), osteogenic (ALP), and myogenic (MYOD, MYOG) markers (Supplementary Figure  2).

### 3.5. Effects on Cell Plasticity

To assess the effects of the Lipogems product on the multidifferentiation capacity of hTSCs, cells preconditioned with the Lipogems product for 96 h were induced to differentiate* in vitro* toward adipocytes and osteoblasts Figures 4(b) and 4(c); see Methods. Results showed no noticeable differences in the adipogenic (Figure 4(b)) and osteogenic (Figure 4(c)) differentiation of hTSCs, suggesting no significant effects of Lipogems treatment on the differentiation capacity towards these cell phenotypes.

### 3.6. Effects on Stem Cell Marker and VEGF Expression

To evaluate the effects of Lipogems treatment on hTSCs stemness, the expression of Nanog, Oct4, and KLF4 was measured by Real-Time PCR and compared to that of control untreated cells (Figure 4(d)). Results revealed no significant differences in stem cell marker expression between Lipogems-treated and control cells. Analysis of the VEGF, a key marker involved in vasculogenesis and angiogenesis, by Real-Time PCR revealed a significantly higher mRNA expression level, about 1.3-fold (*p* < 0.01) in Lipogems-treated cells as compared to control cells (Figure 4(e)).

### 3.7. Effects on Cell Migration

In order to evaluate the effects of the Lipogems product on the repairing capacity of hTSCs, an* in vitro* wound-healing assay was performed. The wound was completely closed in all conditions within 45–48 h, and Lipogems-treated and control cells showed a similar rate of wound closure (a representative image of Lipogems-treated and control hTSCs moving into the wound space is shown in (Figure 4) for both groups at 0 and 25 h after scratching). Quantitative analyses indicated no significant differences in cell migration velocity between Lipogems-treated and control cells at all time points (*p* < 0.05, Figure 4(f)).

## 4. Discussion

The stromal vascular fraction, which is mainly responsible for growth-factors production and regulation, has been successfully used in orthopedic practice [19, 29–31]. However, a precise chemical identification of these factors is still missing. Nonetheless, many reports support the notion that a defined combination of these molecules could greatly improve rotator cuff tendon healing via autocrine and paracrine signaling [16, 17]. For instance, according to the results recently reported by Doornaert et al., it is likely that the lipoaspirate provides local signals to stimulate the self-healing processes by activating resident stem cells through paracrine mechanisms [16]. The recent discovery that also the human rotator cuff possesses a population of resident progenitor stem cells [22, 23], that can be easily isolated and cultured* in vitro*, allowed the design of this study, with the ultimate goal of better understanding the effects of the Lipogems product on adult stem cells. A key question that needed to be addressed was to ascertain whether the beneficial effects of the Lipogems product were due to soluble factors released by the lipoaspirate. Therefore, the use of a transwell system allowed studying the effects of Lipogems product without a direct contact between the lipoaspirate and the stem cell population. These types of indirect coculturing systems have been previously used to successfully direct the differentiation of stem cells and to promote cell survival and expansion* in vitro* [32]. Thus, in all reported experiments, we could assume that the observed effects were due to soluble factors released in the medium by the lipoaspirate. Results showed that hTSCs cocultured with the Lipogems product significantly increased their proliferation capability, with no appreciable cytotoxic effects. Indeed, finding new ways to activate stem cells is vital for an effective treatment, as stem cell progenitors are usually present in very low numbers in adult tissues. Attempts to circumvent the problem by harvesting adult progenitors from biopsies, expanding them* in vitro*, and then reinjecting into the patients have many drawbacks. In fact, isolating and culturing stem cells at GMP level are very troublesome and expensive. Moreover, to date, all methods for culturing stem cells* in vitro* cannot prevent their tendency to lose potency during passages and eventually to become senescent. Therefore, local injection of products like Lipogems could represent a more practical way of activating resident stem cells without the need to isolate them from biopsies. Our results are in agreement with those reported on Human Umbilical Vein Endothelial Cells (HUVECs), where a marked proliferation increase by Lipogems product addiction to the cultures was observed [33]. Interestingly, in our study, we did not observe any loss of stemness when hTSCs were cultured in the presence of Lipogems product, as no significant changes in key stem cells markers could be observed. Moreover, treatment with Lipogems product alone did not induce tenogenesis, adipogenesis, osteogenesi, nor myogenesis. These findings support the notion that the lipoaspirate releases factors that increase stem cell proliferation without altering their potency. These results were confirmed also by differentiation experiments, where Lipogems-treated hTSCs could be induced to differentiate towards osteoblasts and adipocytes with the same efficiency as untreated controls.

Finally, we observed that Lipogems exposure moderately increases hTSC expression of VEGF, which has been shown to play a central role in the neovascularization process, being the main regulator of angiogenesis [17, 34–39]. While at this stage we cannot predict whether the observed VEGF increase could have biological effects, angiogenesis is to be considered an essential factor in designing novel approaches for tissue engineering, as the restoration of blood flow would provide a source of nutrients and oxygen and metabolic substrates and also the access for circulating cells that can help supporting tissue regeneration [19, 40]. Although no studies have directly evaluated the role of VEGF in rotator cuff repair, some reported results demonstrate an improved tensile strength in animal tendon healing models with VEGF augmentation [17, 41]. Moreover, increase in VEGF mRNA expression levels in a rat model of supraspinatus tendon overuse injury was reported [42]. Clearly, at this stage, it cannot be excluded that the Lipogems product releases paracrine angiogenic and antiapoptotic factors other than VEGF. It has been documented that intact human adipose stem cells (hASCs), which can be found in the Lipogems product, are significantly more responsive than enzymatically dissociated hASCs to a mixture of hyaluronic, butyric, and retinoic acid, previously shown to induce stem cell expression of several vasculogenic genes. Therefore, the peculiarity and effectiveness of the Lipogems product may reside in the easily obtainable source of living hASCs that, once injected, could release a variety of soluble factors able to activate resident hTSCs.

## 5. Conclusions

Herein we reported the effects of the Lipogems lipoaspirate on human tendon stem cells from the rotator cuff. Coculture of these progenitor cells with Lipogems released factors significantly increases cell proliferation, without affecting stem cell marker expression and differentiation capability. Overall, these results support that activating resident progenitor cells with the Lipogems product, without the need to isolate stem cells from a biopsy, expanding them* in vitro*, and then re-injecting them into the patient, could be a practical and cost-effective new therapeutic approach for increasing rotator cuff tendon healing. Although the Lipogems product released factors have yet to be identified, preliminary results in our laboratories revealed that the effects are not limited to tendon stem cells. In fact, preliminary increase in proliferation could be observed also on stem cells from the cardiovascular system (data not shown). Therefore, understanding the chemical composition of the Lipogems product could allow chemical synthesis of these factors and using them instead of the lipoaspirate. Further studies in this direction are currently ongoing in our laboratories.

## Supplementary Material

Amplification efficiency of S14 primers by Real-Time PCR.

## Figures and Tables

**Table 1:** Summary of the surface markers by cytofluorimetry of Lipogems-treated and control hTSCs. Numbers show the expression percentage for each cell surface protein.

Markers	hTSCs (control) (%)	hTSCs + Lipogems (%)
CD9	55.31	65.91
CD13	100	100
CD18	0	0
CD29	98.27	96.77
CD34	0	0
CD44	100	100
CD45	0	0
CD71	62.28	37.25
CD73	100	100
CD90	100	100
CD105	100	100
CD106	3.98	2.30
CD117	0	0
CD140a	0	0
CD140b	47.15	3.46
CD146	1.35	0
CD166	84.72	86.99
HLA-ABC	100	100
HLA-DR	0	0
Lineage	0.23	0
NG2	0	0
SSEA-4	0	0
Stro-1	0	0
