# Supplementary material for: Lipogems Product Treatment Increases the Proliferation Rate of Human Tendon Stem Cells without Affecting Their Stemness and Differentiation Capability
Source: Stem Cells Int. 2016 Jan 6;2016:4373410. doi: 10.1155/2016/4373410 (PMC4736573; doi:10.1155/2016/4373410)

Supplementary Material

Supplementary Material provided online contains the amplification efficiency of the reference gene S14 (Suppl. Figure 1), and the adipogenic, osteogenic and myogenic marker expression data measured by Real-Time PCR at 96 h of treatment with the Lipogems® product (Suppl. Figure 2).

Supplementary Figure 1: Amplification efficiency of the reference gene S14 measured by Real-Time PCR. A typical result is shown.

Supplementary Figure 1: Effects of the Lipogems® product on adipogenic, osteogenic and myogenic differentiation promotion. (A) Adipogenic (PPAR-γ and LPL), osteogenic (ALP), and (B) myogenic (MYOD, MYOG) marker expression by Real-Time PCR at 96 h of treatment with the Lipogems® product. Values are expressed as fold-changes relative to control cells. Data are expressed as the means ± SD of three different experiments. p-values were calculated using T student test. Only p-values <0.05 are indicated. N/E = not expressed.

Supplementary Figure 1

Amplification efficiency of human ribosomal protein S14 in Real-TIme PCR

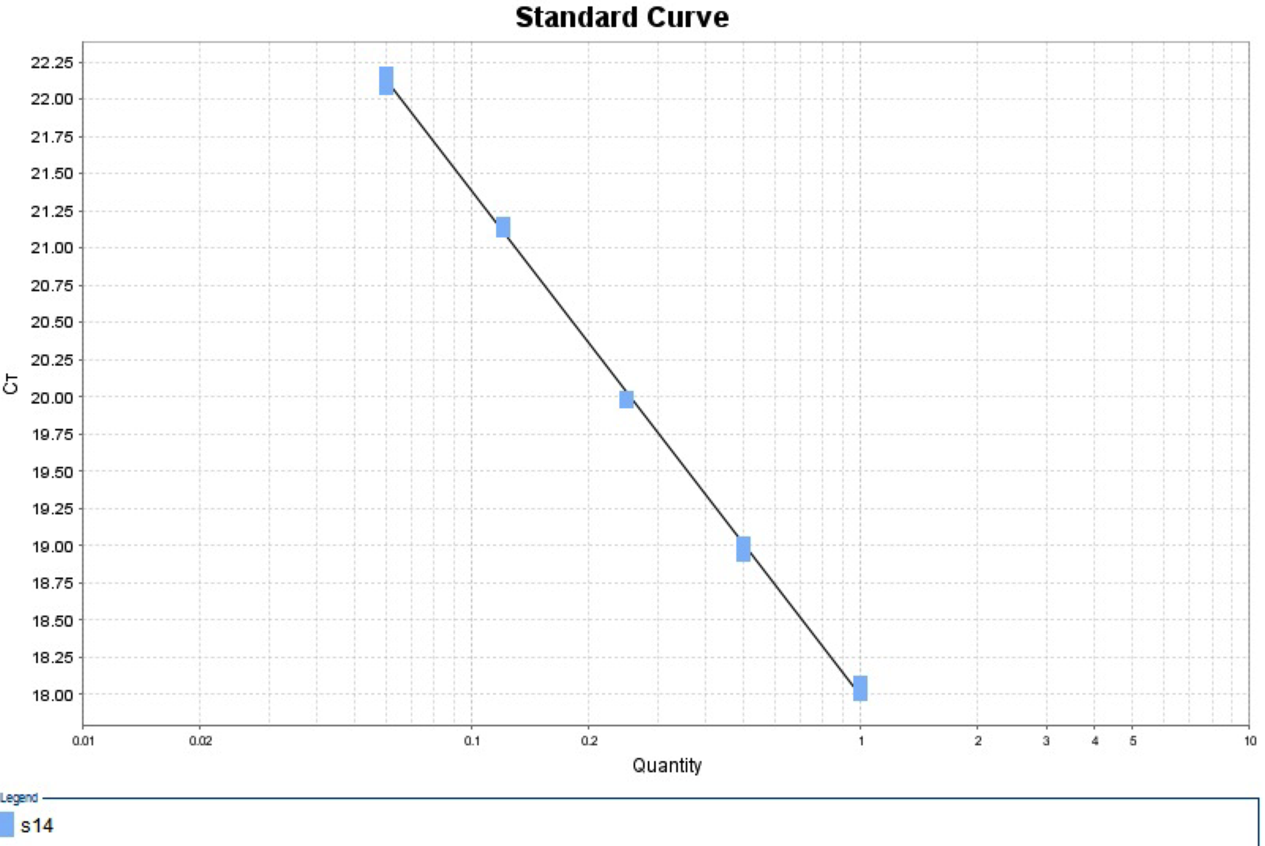

R2 = 0.999; Eff% = 97.8; Slope = -3.376

Supplementary Figure 2

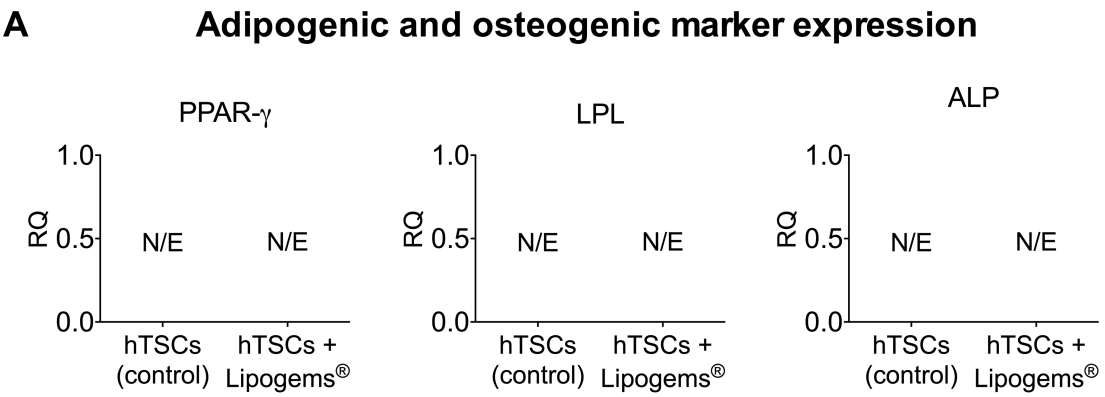

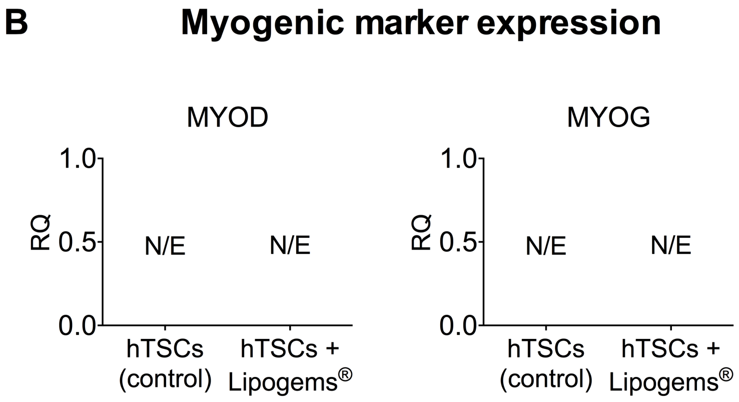

Supplement: Supplementary file 1 — Amplification efficiency of S14 primers by Real-Time PCR. [file 4373410.f1.pdf]
